# Control of Early Blight Fungus (*Alternaria alternata*) in Tomato by Boric and Phenylboronic Acid

**DOI:** 10.3390/antibiotics11030320

**Published:** 2022-02-28

**Authors:** Katarina Martinko, Siniša Ivanković, Boris Lazarević, Edyta Đermić, Damir Đermić

**Affiliations:** 1 Department of Plant Pathology, Division of Phytomedicine, Faculty of Agriculture, University of Zagreb, 10000 Zagreb, Croatia; kmartinko@agr.hr; 2 Division of Molecular Medicine, Ruđer Bošković Institute, 10000 Zagreb, Croatia; sivankov@irb.hr; 3 Department of Plant Nutrition, Division of Agroecology, Faculty of Agriculture, University of Zagreb, 10000 Zagreb, Croatia; blazarevic@agr.hr; 4 Division of Molecular Biology, Ruđer Bošković Institute, 10000 Zagreb, Croatia

**Keywords:** plant disease, antimicrobial effect, prophylaxis, antifungal activity, sustainable management

## Abstract

Finding a suitable alternative to the small pool of existing antifungal agents is a vital task in contemporary agriculture. Therefore, intensive research has been conducted globally to uncover environmentally friendly and efficient agents that can suppress pathogens resistant to the currently used antimycotics. Here, we tested the activity of boric acid (BA) and its derivative phenylboronic acid (PBA) in controlling the early blight symptoms in tomato plants infected with pathogenic fungus *Alternaria alternata*. By following the appearance and intensity of the lesions on leaves of the tested plants, as well as by measuring four selected physiological factors that reflect plant health, we have shown that both BA and PBA act prophylactically on fungal infection. They did it by reducing the amount and severity of early blight symptoms, as well as by preventing deterioration of the physiological traits, occurring upon fungal inoculation. Phenylboronic acid was more efficient in suppressing the impact of *A. alternata* infection. Therefore, we conclude that BA, and even more so PBA, may be used as agents for controlling early blight on tomato plants, as they are both quite effective and environmentally friendly.

## 1. Introduction

The basic problem in modern plant production is a very poor choice of available agents for control of phytopathogenic fungi, as well as development of fungal resistance to the agents in use. The problem is being addressed by research to identify environmentally friendly chemicals with as high an antibiotic efficacy as possible.

One such chemical may be phenylboronic acid (PBA), a phenyl derivative of medicinally important boric acid (BA) [1,2]. Among other sources, boric acid can be found in plants, including almost all fruits [3]. Phenylboronic acid is a commercially available and pharmaceutically acceptable substance [4] that in certain concentrations has a fungicidal effect on several species of human fungi [5], is not toxic to the environment [6] and mammals [7] and has an antitumor effect [7].

Phenylboronic acid is known to interact with the membranes of various fungal cells through the carbohydrates that make up the cell membrane [8,9]. Surprisingly little is known of BA’s mode of action, although it is a commonly used antimicrobial agent in medicine. It affects hyphal transformation and biofilm development, but the inhibition of the oxidative mechanism appears to be its key antifungal mechanism [10]. Toxic chemical compounds based on boron might develop intracellularly; this is suggested by the existence of a boron-containing antibiotic—boromycin, a naturally occurring substance isolated from *Streptomyces* [11]. The study by Liu et al. [4] investigated the fungicidal effect of PBA on two fungal causes of wood decay, *Trametes versicolor* (Linnaeus et Fries) Pilat and *Postia placenta* (Fries) M. Larsen et Lombard [4], where PBA proved to be a potent agent for protecting wood from basidiomycete fungi that cause wood decay. In Yalinkilic et al. [6], the fungicidal efficacy of PBA on fungi *Coriolus versicolor* (L. ex Fr.) Quel. and *Tyromyces palustris* (Berk. and Curt) Murr, which cause a wood decay, was significant. Also, the antifungal effect of PBA on ascomycete fungal pathogens, such as *Aspergillus niger*, *A*. *terreus*, *Fusarium solani*, *F*. *dimerum*, and *Penicillium ochrochloron* was investigated by the agar diffusion method [12]. However, at the used concentrations of 0.01% PBA, no antifungal activity was observed [13]. On the other hand, the results of an earlier in vitro study by Bonnen and Hammerschmidt [14] suggested that PBA at a concentration of 0.01% inhibits the enzyme cutinase in the phytopathogenic fungus *Colletotrichum lagenarium*, which is responsible for degradation of the polymeric part of the plant cuticle.

Ascomycete fungi are the main pathogens in diseases of tomato, which is an important vegetable around the world because many countries have adopted it as a valuable part of their culinary traditions [15]. Because tomato (*Solanum lycopersicum* L.) is grown worldwide for local use or as an export crop, it is not surprising that global economic tomato production is declining due to susceptibility to various pathogens [16]. Tomato cultivation is known to be difficult due to damage caused by pathogens resistant to agrochemicals, including the ascomycete fungus *Alternaria alternata* (Fr.) Keissler [17], which causes early blight [15,18]. It is one of the most important tomato diseases and causes significant losses and reduces the nutritional value of tomato crop. This disease is most harmful to tomato in regions with high humidity and fairly high temperatures in semi-arid climates where frequent and prolonged night dews are common [19]. In plantations in the USA, Australia, Israel, the UK, Brazil, and India, losses caused by this pathogen range from 35 to 80% [20]. In a study by Bessadat et al. [20] the causative agent of the genus *Alternaria* was detected in more than 80% of samples of infected plant material and in more than 50% of total fungal isolates from the plant samples, which confirms the prevalence of this genus. High infestation with early blight leads to complete defoliation and large crop losses in a short period of time [20]. Tomato is infected with *Alternaria* sp. throughout its growth and development, and the infection is characterized by the appearance of chlorotic and necrotic symptoms on the aboveground parts of plant [21]. Economically important symptoms include lesions of basal stems on seedlings, stem lesions on adult plants, and fruit rot [19,22], which reduce yield up to 79%, while lesions on stems can cause seedling losses of 20% to 40% in the field [19,23].

Based on the stated economic importance, control of *A. alternata* is mainly carried out using fungicides to reduce losses [20]. However, there is a consumer concern about pesticide residues in food. New biological control methods for *Alternaria* species include biological methods [24,25] as an alternative to synthetic fungicides to control early blight, as well as the use of environmentally friendly compounds in low concentrations. Accordingly, the aim of this study was to test the effect on *A. alternata* of preventive applications of PBA and BA on tomato in greenhouse conditions. For that, analyses of some physiological parameters of treated tomato plants were performed and the severity of early blight symptoms were assessed.

## 2. Results

### 2.1. BA Effect on Tomato Plants

**Figure 1:**
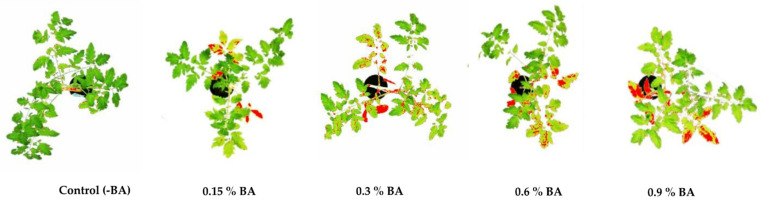
Total physiological lesion area on tomato plants 14 days after BA application, measured by software ImageJ.

The total area of physiological lesions was recorded on the plants onto which various concentrations of the BA were applied (Table 1; Figure 1). Compared to the control (plants treated with no BA), test plants treated with 0.15% and 0.3% BA developed 10% and 22% of the physiological lesion area, respectively, while the test plants onto which 0.6% and 0.9% was applied, showed 34% and 44% of lesion area, respectively. The mean physiological lesions-surface area on the plants treated with BA was significantly higher than that on control plants that received no BA (Tukey’s test, *p* < 0.05) (Table 1).

Our results show (Table 2) that all four assessed parameters did not change significantly in plants 15 days after treatment with BA (Tukey test, *p* < 0.05), as compared to the control (plants without BA application), suggesting that BA treatment in the tested concentration range does not affect general condition of the tested plants.

### 2.2. PBA Effect on Tomato Plants

**Figure 2:**
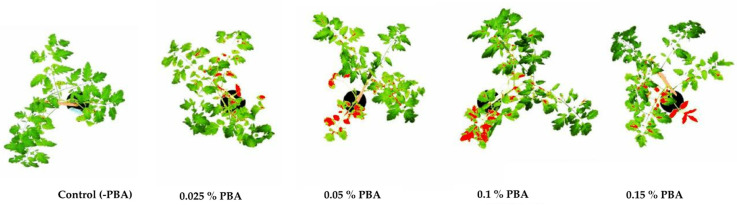
Total physiological lesion area on tomato plants 14 days after PBA application, measured by software ImageJ.

The results of the PBA application on tomato plants, compared to the untreated control, after 14 days are shown in Table 3 and Figure 2. The physiological lesion area was recorded on plant leaves onto which the PBA was applied. Test plants treated with PBA in concentrations 0.025% and 0.05%, showed 7% and 25% of the lesion area, respectively, while those treated with 0.1% and 0.15% PBA developed 31% and 29% of lesion area, respectively. The mean measured physiological lesion areas were significantly higher (Tukey test, *p* < 0.05) than those on the control plants with no PBA applied (Table 3).

The measured physiological parameters of plants treated with PBA, compared to the control variant 15 days post-application, are shown in Table 4. Analogously with BA application, mean values of all tested parameters (CHI, ARI, HUE, NIR) in plants treated with PBA were not significantly different from the parameters in the untreated control (Tukey test, *p* < 0.05). These results indicate that PBA does not significantly affect the general condition of treated plants.

### 2.3. Antifungal Effect of BA on Alternaria alternata

**Figure 3:**
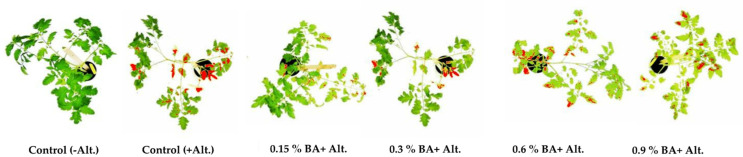
Early blight symptoms on tomato plants pretreated with BA, measured by software ImageJ 14 days after inoculation with *Alternaria alternata*.

The results show the antifungal effect of BA, as reflected in the severity of the symptoms of early blight on inoculated tomato plants, compared to the positive control (plants inoculated with the pathogen and no BA applied) 14 days post-inoculation (Figure 3, Table 5). Relative to the control, test plants treated with BA in concentrations 0.15% and 0.3% developed 17% and 32% of the area with symptoms, respectively, while the plants treated with 0.6% and 0.9% developed 79% and 92% the area of the symptom, respectively. The mean values of symptom area of tomato plants to which BA was applied at concentrations 0.15%, 0.3% and 0.6% of BA, were significantly lower, while the mean value of lesion area of plants treated with BA at a concentration of 0.9% was not significantly lower compared to the affected area in the control (Tukey test, *p* < 0.05).

The assessed physiological parameters of tomato plants inoculated with *A. alternata* are shown in Table 6. It is noticeable that inoculation with the fungus significantly affected (degraded) the parameters (Tukey test, *p* < 0.05). Interestingly, the pretreatment of inoculated plants with BA resulted in tested physiological parameters being very similar to the values of the uninoculated plants, suggesting that BA pretreatment prevents the deleterious impact of fungal infection of the assayed tomato plants. The mean ARI and CHI values of the test plants to which BA was applied in concentrations of 0.15% and 0.9%, were significantly higher, almost twice compared to the analogous values of the positive control, while the mean values of the same parameters on variants with 0.3% and 0.6% were not significantly changed relative to the control (Tukey test, *p* < 0.05). The mean values of HUE in the variants with concentrations 0.15%, 0.3% and 0.9% BA, were significantly increased compared to the control, but in variants with 0.6% BA it was not significantly different from the control. Mean NIR values of the plants treated with all tested concentrations of BA were not significantly increased compared to inoculated plants not treated with BA (Tukey test, *p* < 0.05), yet were lower, though not significantly, than those of the uninoculated plants (Tukey test, *p* < 0.05).

### 2.4. Antifungal Effect of PBA on Alternaria alternata

**Figure 4:**
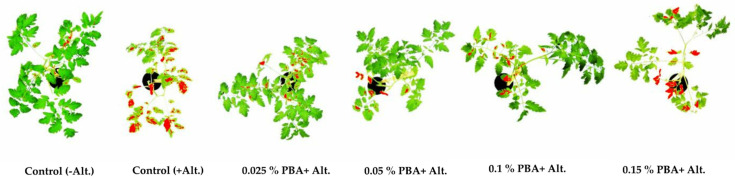
Early blight symptoms on tomato plants pretreated with PBA, measured by software ImageJ 14 days after inoculation with *Alternaria alternata*.

Test plants pretreated with 0.025% and 0.05% PBA developed about 10-fold and 5.5-fold smaller areas of early blight symptoms, respectively, compared to the control treated with no PBA (Figure 4, Table 7), which is significant (Tukey test, *p* < 0.05). The prophylactic effect of PBA pretreatment was weaker in test plants that received 0.1% and 0.15% PBA, their symptoms area was about 3- and 2-fold smaller, respectively, than that of the untreated control (Figure 4, Table 7), but the differences are still significant (Tukey test, *p* < 0.05).

Analogous to the data with BA (Table 6), PBA pretreatment of tomato plants inoculated with *A. alternata*, resulted in the tested physiological parameters being at the level of plants that were not inoculated (Table 8). Mean values of CHI, ARI and HUE parameters were significantly, about 2-fold for the former two, increased in test plants pretreated with PBA in the whole concentration range, compared to the inoculated, but not PBA treated control (Tukey test, *p* < 0.05) (Table 8). The mean NIR values of the plants pretreated with PBA and then infected with the fungus were in between those of uninfected plants and the ones infected but not treated with PBA. These results suggest that pretreatment of tomato plants with PBA mostly annuls the effect of *A. alternata* infection on the assessed physiological factors, i.e., PBA has prophylactic activity.

## 3. Discussion

In this study, we have shown the prophylactic effect of BA and PBA on tomato plants inoculated with *A. alternata*. This was revealed by determining the intensity of early blight symptoms, such as lesion area, as well as physiological parameters that reflect plant health condition. Although both BA and PBA act prophylactically, there were nevertheless notable differences between them. Namely, PBA was effective in lower (about 6-fold) concentrations, the side effects of its application were milder (e.g., the physiological lesion area was smaller), and the protective effect on treated infected plants was stronger (from ~90% to ~60% reduction in the lesion area at the applied concentration range) than that of BA (from ~80% to ~10% reduction in the lesion area at the applied concentration range).

The observed physiological lesion areas in plants treated with BA and PBA (without pathogen inoculation) suggest an adverse effect of the applied agents on the plants’ health, while at the same time those plants showed minimal alteration of the assessed physiological parameters, indicating their good condition. The apparent discrepancy can be explained by taking into consideration that the observed lesions were mostly mild ones, that decrease the chlorophyll level, i.e., chloroses. The software ImageJ is very sensitive and registers even slight depigmentation. However, the observed restricted areas of slight depigmentation, chloroses, did not have any significant effect on the chlorophyll index (CHI) and colour appearance parameter (HUE), representing total chlorophyll content of the test plants, which were unaffected by BA or PBA treatments (Table 2 and Table 4). These data indicate that either BA or PBA application on tomato plants is not deleterious for them in the concentration range we used. Foliar application of boron, most often in the form of BA, can lead to the appearance of physiological lesions because the concentration of boron increases exclusively in the leaves [26]. Although boron in the form of BA enters the plant by passive diffusion, as it is easily permeable through plant cell membranes, the inefficient transport of this micronutrient occurs when photosynthesis is not allowed [27]. That may explain the observed considerable lesion intensity, along with the lack of physiological changes in boronic acid-treated plants, compared to the control plants. Namely, the foliar application of boronic acids is likely followed by difficulties in boron transport through the plant, which results in physiological lesions due to the retention of high boron concentration in the leaves.

Furthermore, we have noted a paradoxical effect, namely the area of lesions in infected plants treated with (P)BA was smaller than the physiological lesion area in the uninfected plants treated with (P)BA (compare Table 1 and Table 5, as well as Table 3 and Table 7). However, considering that phytohormones are involved in boron transport [28], increased phytohormone levels in response to the pathogenesis processes in the infected plants could facilitate boron mobilization from the leaves and thus alleviate symptoms on the leaves.

In the experiments with *A. alternata* inoculation, the pretreatment with either BA or PBA resulted in a significantly reduced area and severity of early blight symptoms in the test plants, indicating the agents’ prophylactic, antifungal activity. An analogous effect was noted when assessing the effect of BA or PBA on the tested physiological traits of the infected plants. The decrease in levels of anthocyanins (ARI), chlorophyll (CHI, HUE) and NIR in the plants infected with *A. alternata*, which indicate their poor health status, was effectively prevented by pretreatment with either BA or PBA (Table 6 and Table 8).

The use of phenotyping techniques with multispectral photographs of plants, provides insight into the physiological background of tomato responses to the infection and application of BA and PBA. Because anthocyanins are nonphotosynthetic pigments associated with plant resistance to stress caused by pathogen attack [29], the anthocyanin content of tomato plants is relevant [30]. Infection of plants with pathogens is known to lead to the synthesis of anthocyanins as short-term protective compounds in plant vegetative tissue [31,32,33,34,35], resulting in increased anthocyanin content [36,37,38]. Recent studies have shown that infection of tomato with the fungal pathogen *Botrytis cinerea* [39], especially with more virulent strain of fungus [40], leads to increased anthocyanin synthesis, which positively impacts the defence response of tomato in pathogenesis. Analogously, a substantial increase in anthocyanin content in *Arabidopsis thaliana* after bacterial infection with *Pseudomonas syringae* pv. *tomato*, leads to a reduction in disease symptoms, as revealed by analysis of a combination of multispectral and fluorescent photographs [41]. Furthermore, the accumulation of anthocyanins around the site of infection, where they absorb excess light and prevent chlorophyll degradation [42], reduces the frequency and severity of photoinhibition, as well as accelerates photosynthetic recovery of the plant [31,37]. The increase in anthocyanin content that we have observed in infected plants pretreated with BA or PBA indicates the activation of the defence response. Štambuk et al. reported that the value of HUE is proportional to the total chlorophyll content of the plant [43]. Therefore, it is not surprising that in this study a significant increase in HUE parameters was found, which was proportional to the increase in chlorophyll content in the infected plants pretreated with BA or PBA.

Plants are known to induce defence responses using tightly regulated phytohormone networks [44] including salicylic acid, an endogenous signalling molecule that plays a key role in protecting plants from infection by pathogens [44,45,46]. This is evidenced by studies [47,48,49,50,51] that highlight the increase in salicylic acid levels after plant infection with phytopathogens. In addition to limiting pathogenesis, increasing salicylic acid levels alleviates the symptoms of phytotoxicity caused by boron deficiency or excess, as pointed out by Nawaz et al. [52]. In support of this, the study of El-Shennawy and Abd El [46] found that the combination of salicylic acid and BA significantly reduced the severity of early blight symptoms, leading to an increase in chlorophyll content in the tested tomato plants. With the application of boronic acids, the presumed synergy of boron compounds and phytohormone compounds resulting from the induction of the tomato defence response likely led to an increase in chlorophyll content (CHI), and thus colour appearance parameter (HUE), and to an increase in near-infrared reflection parameter (NIR). Interestingly, the increase in NIR parameters refers to healthy plants [53,54]. This parameter is not affected by leaf pigment but is determined by the optical properties of the leaf related to leaf morphology, thickness, water content and light scattering within the leaf. By scattering light within the leaf, light is refracted on the surface of unaffected cells in a healthy leaf, making the NIR value higher. On the other hand, pathogen attack damages plant cells, hence light is less refracted within the leaves, resulting in a decrease in NIR values [55].

## 4. Materials and Methods

The antifungal effects of BA (Sigma-Aldrich, St. Louis, MO, USA, CAS 10043-35-3) and PBA (Sigma-Aldrich, USA, CAS 98-80-6) were investigated in greenhouse according to the modified method of Nashwa and Abo-Elyousr [56]. Testing was performed in tomato cv. Rutgers in the postembryonic phenophase (BBCH 109), which marks the appearance of the 9th tomato leaf according to Meier [57].

The experiment was set up in 3 replicates with a total of 72 tomato plants. Plants were grown in optimal conditions for growth and development according to Shamshiri et al. [58]. Seeds were sown in plastic jars (Ø 10 cm) containing ecological substrate (Substral Naturen Bio, Salzburg, Austria). Watering was carried out manually, once a day. Fertilization of tomato plants was carried out according to recommendations of the manufacturer of organic liquid fertilizer for tomatoes (Celaflor Naturen, Salzburg, Austria). Plants were supported by thin wooden sticks.

### 4.1. Preparation of BA and PBA Solutions

In our earlier in vitro study, we determined the minimal concentration of BA and PBA showing inhibitory effect on *A. alternata* using poisoned food technique, according to Qadoos et al. [59]. These data were used to prepare a range of BA and PBA concentrations for this in vivo study.

To make a 1% stock solution, 0.5 mg of BA was dissolved in 50 mL of sterile distilled water. This prepared stock solution was pipetted in a certain volume into sterile distilled water to obtain four final concentrations: 0.15%, 0.3%, 0.6% and 0.9%. The same procedure was applied for PBA to obtain final concentrations: 0.025%, 0.05%, 0.1% and 0.15%. The range of concentrations was prepared in plastic hand sprayers in a volume of 200 mL, sufficient for the application of all variants in an in vivo experiment.

### 4.2. Preparation of Suspension of A. alternata Spores

*Alternaria alternata* was isolated from tomato (cv. Rutgers) leaves with early blight symptoms collected from open field in Gornji Laduč (NW from Zagreb) during summer 2021. The isolate was grown on potato dextrose agar (PDA, Sigma-Aldrich, USA) at 26 °C under white light at 16/8 h day/night regime in a growth chamber for 7 days to stimulate fungal sporulation [60]. The obtained pure culture of the isolated fungus was characterized morphologically [60] and molecularly by the PCR [61] to the species level using primer pair ITS1/ITS4 specific for rDNA region [62]. The amplicons were sequenced (Macrogen Europe, Amsterdam, The Netherlands), and the sequence was deposited at GenBank and they shared 100% homology with *A. alternata* MT482506 (GenBank). A suspension of pathogen spores was prepared from pure 7-day-old cultures by adding 20 mL of sterile distilled water on a developed colony of *A. alternata* in Petri dish, and the fungal growth was scraped off with the help of a laboratory spatula. The number of *A. alternata* spores was measured with a haemocytometer and the suspension of 8.2 × 10^5^ spores·mL^−1^ was poured into a pre-sterilized hand sprayer.

### 4.3. Implementation of the Experiment In Vivo

The range of BA and PBA concentrations, described in 4.1., was applied in the experiment. To test the antifungal effect, preventive application of PBA was carried out 2 days prior to inoculation by foliar spraying of plants with a hand sprayer. For comparison, different concentrations of BA or distilled water were applied on plants 2 days prior to their inoculation. A separate control group contained plants that were treated with analogous concentrations of PBA or BA, without inoculation with the pathogen. For inoculation purposes, tomato plants were wounded using carborundum powder in suspension with *A. alternata* according to Fallik et al. [63], which were applied by a hand sprayer. After inoculation, the tomato plants were covered with plastic foil for 48 h to achieve high humidity, after which the plants were kept in optimal greenhouse conditions (25 ± 2 °C) until the first symptoms appeared.

Readings of the results were performed on the 14th day post-inoculation by computer processing of photographs of plant overhead view with symptoms of the disease, which assessed the severity of symptoms of infected plants (e.g., chlorosis and necrosis) using the computer program ImageJ (open source from U.S. National Institutes of Health, Bethesda, MD, USA [64]) according to Laflamme et al. [65].

In addition to measuring the area of lesions on the treated tomato plants, we assessed the following physiological factors that are indicative of plant health: anthocyanin index (ARI) is associated with plant resistance to stress caused by pathogen attack [29]; chlorophyll index (CHI) and related colour appearance parameter (HUE) evaluate the chlorophyll content of the plant and are thus indicative of the overall plant health, whereas near-infrared reflection (NIR) is not affected by leaf pigment but rather by some biochemical and biophysical properties of the plant cells and therefore also reflects plant health. These physiological parameters were assessed on the 15th day post-inoculation by taking high resolution multispectral photographs of six representative detached leaves using the CropReporter^TM^ (PhenoVation B.V., Wageningen, The Netherlands).

### 4.4. Statistical Analysis

The results of in vivo experiments are presented by their mean values and standard deviations. The mean values were compared by one-way analysis of variance (ANOVA), and differences between treatments were evaluated by Tukey’s test (comparison of significantly different mean values on significance level of *p* < 0.05) [66] in SPS (ver. 27; IBM SPSS Statistics, New York, NY, USA) [67].

## 5. Conclusions

In summary, we have shown here that both BA and PBA have prophylactic activity against *A. alternata* infection of tomato plants, with PBA having several advantages over BA. Considering the scarcity of available antifungal agents, as well as their adverse environmental impact (e.g., copper and its derivatives), a possible environmentally friendly alternative is certainly more than welcome. In that respect we plan to test the curative activity of the two boronic acids against *A. alternata* infection of tomato plants. Also, the formulation of the two agents would be worked on, as well as on the optimization of the application of the agent on plants.

## Figures and Tables

**Table 1:** Effect of BA on tomato plants, 14 days after application.

BA Volume Concentration (%)	0.0	0.15	0.3	0.6	0.9
Mean value of physiological lesion area * ± SD	0 ^a^ ± 0	9.5 ^b^ ± 1	22.3 ^c^ ± 2.1	34.4 ^d^ ± 1.4	44.4 ^e^ ± 2.2

* Different letters indicate a statistically significant difference according to the Tukey test (*p* < 0.05).

**Table 2:** Effect of BA on physiological parameters of tomato plants, 15 days post-application.

BA Concentration (%)	ARI Mean Value of Lesion Area ± SD	CHI Mean Value of Lesion Area ± SD	HUE Mean Value of Lesion Area ± SD	NIR Mean Value of Lesion Area ± SD
0	2.46 ± 0.7	1.88 ± 0.4	99.3 ± 4.7	18,329.5 ± 1112.2
0.15	2.77 ± 0.9	1.96 ± 0.5	100.7 ± 8.7	18,203.5 ± 491.8
0.3	2.49 ± 1	1.81 ± 0.5	98.3 ± 8.4	18,832.1 ± 1112.5
0.6	2.27 ± 0.9	1.68 ± 0.6	95.3 ± 12.1	18,173.5 ± 937.8
0.9	2.11 ± 1	1.67 ± 0.5	95.9 ± 8.5	17,485.8 ± 1029.0

There were no significant differences between the concentrations (Tukey test, *p* < 0.05).

**Table 3:** Effect of PBA on tomato plants, 14 days after application.

PBA Concentration (%)	0.0	0.025	0.05	0.1	0.15
Mean value of physiological lesion area * ± SD	0 ^a^ ± 0	6.7 ^b^ ± 0.7	24.7 ^c^ ± 2.3	31 ^d^ ± 3.3	29.2 ^cd^ ± 2.3

* Different letters (^a–d^) indicate a statistically significant difference according to the Tukey test (*p* < 0.05).

**Table 4:** Effect of PBA on some physiological parameters of tomato plants 15 days post-application.

PBA Concentration (%)	ARI Mean Value of Lesion Area ± SD	CHI Mean Value of Lesion Area ± SD	HUE Mean Value of Lesion Area ± SD	NIR Mean Value of Lesion Area ± SD
0	2.46 ± 0.4	1.88 ± 0.9	99.3 ± 4.7	18,329.5 ± 1112.2
0.025	2.34 ± 0.5	1.91 ± 0.7	100.3 ± 7.7	18,201.7 ± 440.6
0.05	2.71 ± 0.5	2.03 ± 0.8	102.1 ± 5.8	18,351.3 ± 507.1
0.1	2.48 ± 0.5	1.90 ± 0.9	99.6 ± 7.4	18,302.7 ± 757.1
0.15	2.68 ± 0.5	1.95 ± 1.0	100.3 ± 7.0	18,158.3 ± 643.6

There were no significant differences between the concentrations (Tukey test, *p* < 0.05).

**Table 5:** Effect of tomato plants pretreatment with BA on the severity of early blight symptoms, caused by *Alternaria alternata* 14 days post-inoculation.

BA Concentration (%)	0.0	0.15	0.3	0.6	0.9
Mean value of symptoms area * ± SD	29.8 ^d^ ± 0.9	5 ^a^ ± 1.1	9.5 ^b^ ± 0.5	24 ^c^ ± 1.8	27.7 ^d^ ± 1.4

* Different letters indicate a statistically significant difference according to the Tukey test (*p* < 0.05).

**Table 6:** Physiological parameters of tomato plants pretreated with BA and inoculated 2 days later with *Alternaria alternata*, and measured 15th day post-inoculation.

BA Concentration (%)	ARI Mean Values of Symptoms * ± SD	CHI Mean Values of Symptoms * ± SD	HUE Mean Values of Symptoms * ± SD	NIR Mean Values of Symptoms * ± SD
0	2.46 ^a^ ± 0.4	1.88 ^a^ ± 0.9	99.3 ^a^ ± 4.7	18,329.5 ^a^ ± 1112.2
0 + Alt.	1 ^a^ ± 0.5	0.9 ^a^ ± 0.4	80.2 ^a^ ± 10.3	16,404.0 ^a^ ± 1199
0.15 + Alt.	2.1 ^b^ ± 0.5	1.7 ^b^ ± 0.3	96.7 ^b^ ± 4.9	17,250.6 ^a^ ± 618.8
0.3 + Alt.	1.7 ^ab^ ± 0.4	1.5 ^ab^ ± 0.3	93.6 ^b^ ± 4.1	17,414.1 ^a^ ± 410.7
0.6 + Alt.	1.7 ^ab^ ± 0.7	1.4 ^ab^ ± 0.4	91.3 ^ab^ ± 9.5	17,160.6 ^a^ ± 723.7
0.9 + Alt.	2.1 ^b^ ± 0.6	1.7 ^b^ ± 0.3	96.4 ^b^ ± 6.3	17,336.6 ^a^ ± 426.4

* Different letters (^a,b^) indicate a statistically significant difference according to the Tukey test (*p* < 0.05).

**Table 7:** Effect of tomato plants pretreatment with PBA on the severity of early blight symptoms, caused by *Alternaria alternata* 14 days post-inoculation.

PBA Concentration (%)	0	0.025	0.05	0.1	0.15
Mean value of symptoms area * ± SD	29.8 ^e^ ± 0.9	2.8 ^a^ ± 0.2	5.5 ^b^ ± 0.5	9.6 ^c^ ± 0.6	17.5 ^d^ ± 1.7

* Different letters indicate a statistically significant difference according to the Tukey test (*p* < 0.05).

**Table 8:** Physiological parameters of tomato plants treated with PBA, 2 days later inoculated with *Alternaria alternata* and measured 15th day post-inoculation.

PBA Concentration (%)	ARI Mean Value of Symptoms * ± SD	CHI Mean Value of Symptoms * ± SD	HUE Mean Value of Symptoms * ± SD	NIR Mean Value of Symptoms * ± SD
0	2.46 ^a^ ± 0.4	1.88 ^a^ ± 0.9	99.3 ^a^ ± 4.7	18,329.5 ^a^ ± 1112.2
0 + Alt.	1.0 ^a^ ± 0.5	0.9 ^a^ ± 0.4	80.2 ^a^ ± 10.3	16,404.0 ^a^ ± 1199.0
0.025 + Alt.	2.2 ^b^ ± 0.6	1.8 ^b^ ± 0.3	98.7 ^b^ ± 4.4	17,504.8 ^ab^ ± 237.1
0.05 + Alt.	2.0 ^b^ ± 0.6	1.7 ^b^ ± 0.3	96.6 ^b^ ± 6.2	17,832.1 ^b^ ± 390.4
0.1 + Alt.	2.2 ^b^ ± 0.6	1.8 ^b^ ± 0.3	99.0 ^b^ ± 5.1	17,450.0 ^ab^ ± 763.6
0.15 + Alt.	2.4 ^b^ ± 0.6	1.8 ^b^ ± 0.3	99.4 ^b^ ± 4.9	17,376.0 ^ab^ ± 719.7

* Different letters (^a,b^) indicate a statistically significant difference according to the Tukey test (*p* < 0.05).

## Data Availability

Not applicable.
